# DisCo P-ad: Distance-Correlation-Based ***p***-Value Adjustment Enhances Multiple Testing Corrections for Metabolomics

**DOI:** 10.3390/metabo15010028

**Published:** 2025-01-08

**Authors:** Debmalya Nandy, Debashis Ghosh, Katerina Kechris

**Affiliations:** 1 Department of Biostatistics & Informatics, Colorado School of Public Heath, University of Colorado Anschutz Medical Campus, Aurora, CO 80045, USA; drdeb@nandy.pro (D.N.); debashis.ghosh@cuanschutz.edu (D.G.); 2 Center for Innovative Design & Analysis, Department of Biostatistics & Informatics, Colorado School of Public Heath, University of Colorado Anschutz Medical Campus, Aurora, CO 80045, USA

**Keywords:** multiple testing, effective number of tests, correlated tests, eigen-analysis, pointwise error rate, metabolome-wide association study

## Abstract

Background: Due to scientific advancements in high-throughput data production technologies, omics studies, such as genomics and metabolomics, often give rise to numerous measurements per sample/subject containing several noisy variables that potentially cloud the true signals relevant to the desired study outcome(s). Therefore, correcting for multiple testing is critical while performing any statistical test of significance to minimize the chances of false or missed discoveries. Such correction practice is commonplace in genome-wide association studies (GWAS) but is also becoming increasingly relevant to metabolome-wide association studies (MWAS). However, many existing procedures may be too conservative or too lenient, only assume a linear association between the features, or have not been evaluated on metabolomics data. Methods: One such multiple testing correction strategy is to estimate the number of statistically independent tests, called the *effective number of tests*, based on the eigen-analysis of the correlation matrix between the features. This effective number is then used for a subsequent single-step adjustment to obtain the pointwise significance level. We propose a modification to the *p*-value adjustment based on a more general measure of association between two predictors, the *distance correlation*, with a specific focus on MWAS. Results: We assessed common GWAS *p*-value adjustment procedures and one tailored for MWAS, which rely on eigen-analysis of the Pearson’s correlation matrix. Our study, including varying sample size-to-feature ratios, response types, and metabolite groupings, highlights the superior performance of the distance correlation. Conclusion: We propose the distance-correlation-based *p*-value adjustment (DisCo P-ad) as a novel modification that can enhance existing eigen-analysis-based multiple testing correction procedures by increasing power or reducing false positives. While our focus is on metabolomics, DisCo P-ad can also readily be applied to other high-dimensional omics studies.

## 1. Introduction

Over the past two decades, advances in technology have led to the generation of big data in the omics fields, including genomics, proteomics, and metabolomics. The high-dimensional nature of these data has posed challenges for the data-scientists in identifying true signals among many potentially noisy features. Ref. [1] presents a literature review listing some common types of data analysis scheme for *multi-omics* studies along with the computational and statistical methods used for each type. To make inferences regarding relationships between features and the outcome(s) of interest, one often performs multiple tests on a per-feature level. Now note that, in every test of hypothesis, one may encounter two types of errors [2]: for a type-I error, we come up with a false signal (*false positive*, i.e., rejecting a null hypothesis when it is actually true); whereas, for a type-II error, we fail to detect a true signal (*false negative*, i.e., fail to reject a null hypotheses when it is actually false). It is important to prefix the maximum allowable type-I error (e.g., 5%), also termed as the *significance level* of a statistical test. However, in multiple testing, to restrain the overall type-I error rate for the family of individual tests (family-wise error rate, FWER), α (probability that at least one null hypothesis is rejected when it is true), we need to consider a corresponding individual test-specific error rate (point-wise error rate, PWER), $\alpha_{f}$, which satisfies the following equation: $\alpha =FWER\leq 1-{\left(1-\alpha_{f}\right)}^{M}$, where *M* is the total number of tests in the family. Notably, $\alpha_{f}\leq \alpha$.

A simplistic and, hence, widely used *p*-value adjustment procedure is the *Bonferroni* correction [3]:

(1) $$\alpha_{f}^{\left(B\right)}=\alpha /M,$$

where *M* tests are assumed to be mutually independent. However, Bonferroni is also the most conservative adjustment, potentially resulting in many false negatives. Ref. [4] proposed an alternative adjustment:

(2) $$\alpha_{f}^{\left(S\right)}=1-{\left(1-\alpha \right)}^{1/M}.$$

Note that Equation (2), when expanded up to the second term in a Taylor series expansion, gives rise to Equation (1). Also, as for the Bonferroni adjustment, the derivation of Šidák’s formula also assumes that all the *M* tests of hypotheses are mutually independent. Unlike controlling for an overall type-I error rate (FWER), another popular approach is to control the expected proportion of falsely identified significant discoveries among all the declared significant ones, termed as *false discovery rate* (FDR; [5,6,7,8,9]).

FDR is a more suitable false discovery controlling measure for datasets with relatively more prevalent signals than those with sparser signals, such as mass-spectrometry metabolomics. Moreover, as pointed out in [10], (a) FDR is effective in controlling the false signals with independent and positively dependent tests and is affected by correlated tests; and (b) FWER offers a tighter control over type-I error rate than the FDR.

In addition to the above procedures, there exists the permutation-based gold-standard approach to estimate $\alpha_{f}$ [11,12]. The main drawback of this approach, however, is the severe computational cost, making this approach practically infeasible to be implemented for large datasets [13,14]. Depending on the size of the dataset (the sample size (*n*) and the total number of features (*M*)) and the overall type-I error rate (α), one often needs to implement several thousand permutations for accurate estimation of $\alpha_{f}$ (e.g., at least 1000 permutation shuffles for α=0.05 and 10,000 for α=0.01; [15]).

Note that the assumption of the statistical tests to be mutually independent is often violated with omics data, e.g., single nucleotide polymorphisms (SNPs) in GWAS are in linkage disequilibrium. See [16] for a review of pros and cons of some popular *p*-value correction methods in quantitative omics experiments. Therefore, as an alternative, one can estimate the effective number of tests, M_eff_, and use that estimate for a Bonferroni- or Šidák-type correction as follows [17]:

(3a) $$\begin{array}{cc} {\hat{\alpha }}_{f}^{\left(B\right)} & =\alpha /{\hat{M}}_{\mathrm{eff}} \end{array}$$

for Bonferroni-type adjustment, or

(3b) $$\begin{array}{cc} {\hat{\alpha }}_{f}^{\left(S\right)} & =1-{\left(1-\alpha \right)}^{1/{\hat{M}}_{\mathrm{eff}}} \end{array}$$

for Šidák-type adjustment.

In the context of genome-wide association studies (GWAS), M_eff_ is estimated based on the eigen-analysis of the features’ correlation matrix, where the features are SNPs with allele counts 0,1, or 2. The most commonly used measure of correlation is the Pearson’s correlation (PrsCo; [18,19,20,21]). In such cases, the estimate ${\hat{M}}_{\mathrm{eff}}$ is a function of the eigen-values obtained from the eigen-analyses and ${\hat{M}}_{\mathrm{eff}}\leq M$ [13,15,17,22,23]. Most methods have focused on GWAS, but there is increasing interest to identify the effective number of tests in the metabolomics context. However, the association structure between metabolites may be different to that found with SNPs. More recently, [10] proposed a new ${\hat{M}}_{\mathrm{eff}}$ approach for the metabolome-wide association studies (MWAS).

Therefore, in order to derive a more realistic and robust ${\hat{M}}_{\mathrm{eff}}$ measure, i.e., one that accounts for both linear and non-linear types of association among the features that are likely in metabolomics data, we propose to apply the *distance correlation* (DisCo; [24]). Note that, *signed distance correlation* has been introduced for the analysis of metabolomic and lipidomic data [25], but not for calculating the effective number of tests, which is the focus of this work. We examine how the ${\hat{M}}_{\mathrm{eff}}$ estimation methods apply to metabolomics data by estimating M_eff_ using DisCo for the above-mentioned eigen-analysis-based methods applied on a metabolomics dataset. By means of simulations and a real data application, we demonstrate the effectiveness of using DisCo compared to PrsCo in reducing potential false negatives and false positives for multiple testing corrections in the metabolomics context. We also consider how results are affected by known groupings of metabolites based on pathway annotations. Although the focus is on metabolomics data, the proposed DisCo-based *p*-value adjustment procedure (DisCo P-ad) can be used for any eigen-analysis-based method to account for both linear and non-linear associations in omics datasets.

## 2. Materials and Methods

### 2.1. Methods to Estimate M_eff_

We compare the performance of seven procedures to estimate M_eff_, six popular in GWAS literature [26] and one recently introduced for MWAS: 1. Bonferroni [3]; 2. Šidák [4]; 3. Nyholt [22]; 4. Li and Ji [23]; 5. Gao et al. [13]; 6. Galwey [17]; and 7. Peluso et al. [10]. For the estimation of M_eff_, for Bonferroni and Šidák, $M_{\mathrm{eff}}=$ *M*; whereas the last five procedures are based on eigen-analysis of the features’ correlation matrix. To obtain eigen-vectors, we use *principal component analysis* (PCA; [27,28]). PCA is a high-dimensional data analysis procedure that provides projected directions of maximum variation among a set of data points in a real coordinate space. In an *M*-dimensional space, the unit-length *principal components* (PCs; directions) are computed sequentially. That is, the *m*-th PC is orthogonal to all the previous (m−1) PCs while explaining the maximum variability not explained by the previous PCs. Thus, PCA provides orthonormal bases with *M* spanning vectors projecting the original data along the directions of maximum variability. Often, researchers use PCA as a tool for reducing the dimension of the *M*-dimensional data to a lower-dimensional space spanned by just the first few PCs. In our context, PCA can be used on the correlation matrix (using PrsCo or DisCo) of the metabolomics features. For example, let *A* be an *M*-by-*M* correlation matrix of *M* metabolites’ abundances (log-transformed and adjusted for the clinical covariates, if applicable). Then the elements of $A=(\left(a_{ij}\right)),i,\;j=1,\ldots ,M,$ will, respectively, consist of the PrsCo or the DisCo of the $i^{th}$ and the $j^{th}$ metabolites’ abundances. In the equations below, ${\hat{\lambda }}_{i}$ are the *M* eigenvalues obtained from the eigen-analysis of the estimated correlation matrix, such that ${\hat{\lambda }}_{1}\leq {\hat{\lambda }}_{2}\leq {\hat{\lambda }}_{3}\leq \ldots \leq {\hat{\lambda }}_{M}$. Note that, while the eigen-values obtained from the eigen-analysis of the PrsCo matrix conveniently indicate the variance explained by the corresponding eigen-vectors, the same interpretation might not necessarily hold true for the DisCo matrix. Our study objective, however, is not to intepret the variance-explaining features of the eigen-values/vectors, but, rather, to use the matrix as an intermediate step for accurately estimating the effective number of tests in a multiple testing scenario.

The five eigen-analysis-based procedures to estimate M_eff_ give rise to the following:

(4a) $$Nyholt:{\hat{M}}_{\mathrm{eff}}^{N}=1+\left(M-1\right)\cdot \left(1-\mathrm{var}\left(\hat{\lambda }\right)/M^{2}\right);$$

(4b) $$LiJi:{\hat{M}}_{\mathrm{eff}}^{\mathrm{LiJi}}=\sum_{i}f(|{\hat{\lambda }}_{i}\left|\right);\mathrm{where},\;f\left(x\right)=I\left(x\geq 1\right)+\left(x-\left\lfloor x\right\rfloor \right);$$

(4c) $$Gao:{\hat{M}}_{\mathrm{eff}}^{G}=\mathrm{no}.\;\mathrm{of}\;\mathrm{PCs}\;\mathrm{explaining}\;\geq 99.5\%\;\mathrm{of}\;\mathrm{total}\;\mathrm{variation};$$

(4d) $$Galwey:{\hat{M}}_{\mathrm{eff}}^{\mathrm{Gw}}={\left(\sum_{i}\sqrt{{\hat{\lambda }}_{i}}\right)}^{2}/\sum_{i}{\hat{\lambda }}_{i};$$

(4e) $$Peluso:{\hat{M}}_{\mathrm{eff}}^{P}={\left(\sum_{i}\sqrt{{\hat{\lambda }}_{i}}/\log({\hat{\lambda }}_{1})\right)}^{2}/\left(\sum_{i}{\hat{\lambda }}_{i}/{\hat{\lambda }}_{1}+\sqrt{{\hat{\lambda }}_{1}}\right).$$

### 2.2. Evaluating the Performance of Estimated $M_{\mathrm{eff}}$

Although it can be computationally expensive for large data (high *n* or *M*), the “gold-standard” is the permutation-based approach, which we used to evaluate the performance of estimated M_eff_ values using the seven procedures described in Section 2.1. For a fixed FWER (α=0.05), we estimated the permutation-based PWER, denoted as ${\hat{\alpha }}_{f}^{0}$. Then, we computed the gold-standard effective number of tests as ${\hat{M}}_{\mathrm{eff}}^{0}=\alpha /{\hat{\alpha }}_{f}^{0}$. Next, we estimated M_eff_ values twice for each of the five eigen-analysis-based procedures—once using the PrsCo matrix and once using the DisCo matrix—and compared those two estimates to ${\hat{M}}_{\mathrm{eff}}^{0}$. The closer the value, the better the estimation performance. Note, however, for Bonferroni and Šidák, ${\hat{M}}_{\mathrm{eff}}$ = *M* always. As a secondary evaluation with the gold-standard approach, inspired by [13], for each of the methods with two association types (PrsCo, DisCo), we also estimated the PWER, ${\hat{\alpha }}_{f}=\alpha /{\hat{M}}_{\mathrm{eff}}$. The closer ${\hat{\alpha }}_{f}$ is to the gold-standard ${\hat{\alpha }}_{f}^{0}$, the better.

### 2.3. Computation of Permutation-Based Gold-Standard $\alpha_{f}^{0}$

We followed the procedure described in [10] and adopted their R-programming code to estimate the gold-standard PWER, $\alpha_{f}^{0}$.

1. Randomly assign the *n* outcomes to *n* samples (subjects), keeping each sample in conjunction with its fixed clinical covariates (if applicable) and *M* metabolite abundances. This way, we generate a dataset under null hypothesis of no association between clinical outcomes and metabolite abundances.
2. Compute *M* linear regression models, using one metabolite at a time and adjusting for the clinical covariates in each model (if applicable).
3. Store the minimum of the *M* *p*-values, i.e., one that corresponds to the highest threshold value that rejects all the *M* null hypotheses.
4. Repeat steps 1–3 *K* times (≥n/2); obtain a vector of length *K*, say q, composed of the *K* minimum *p*-values.
5. Sort q in ascending order. The $\left[\alpha K\right]$-th value indicates the gold-standard PWER estimate, ${\hat{\alpha }}_{f}^{0}$.
6. Finally, estimate the gold-standard M_eff_: ${\hat{M}}_{\mathrm{eff}}^{0}=\alpha /{\hat{\alpha }}_{f}^{0}$.

Note that, at step 5, one can use the Gaussian approximation to a binomial distribution to obtain a desired confidence interval for ${\hat{\alpha }}_{f}^{0}$. For our study, we set the FWER at α=0.05 and the number of permutations at K=10,000.

### 2.4. DisCo P-ad Algorithm

First, we introduce the *distance covariance* and *distance correlation*, originally proposed by [24]. Let X and Y be two random vectors with arbitrary dimensions *l* and *m*, respectively, and finite first-order moments. Also, let $f_{X,Y}$, $f_{X}$, and $f_{Y}$, respectively, denote the characteristic functions of the joint distribution of (X,Y) and the marginal distributions of X and Y. Then, the *distance covariance* between X and Y is the non-negative number, η(X,Y), defined as follows:

(5) $$\begin{array}{c} \eta^{2}\left(X,Y\right)={∥f_{X,Y}\left(s,t\right)-f_{X}\left(s\right)\cdot f_{Y}\left(t\right)∥}^{2}\\ =\frac{1}{c_{l}\cdot c_{m}}\int_{R^{l+m}}\frac{|f_{X,Y}\left(s,t\right)-f_{X}\left(s\right)\cdot f_{Y}{\left(t\right)|}^{2}}{{\left|s\right|}^{1+l}\cdot {\left|t\right|}^{1+m}}\cdot dt\;ds, \end{array}$$

where $c_{l}$ and $c_{m}$ are two scalars that are functions of *l* and *m*, respectively, and ∥γ(u,v)∥ denotes the norm of the complex function γ(·,·) defined on $R^{l}\times R^{m}$. Following Equation (5), the *distance variance* of a random vector, say X, can be defined as follows:

(6) $$\eta^{2}\left(X\right)=\eta^{2}\left(X,X\right)={∥f_{X,X}\left(s,t\right)-f_{X}\left(s\right)\cdot f_{X}\left(t\right)∥}^{2}.$$

Finally, using the definitions of distance covariance and distance variance in Equations (5) and (6), we define the *distance correlation* (DisCo) as the non-negative number R(X,Y)∈[0,1] as follows:

(7) $$R^{2}\left(X,Y\right)=\left\{\begin{array}{cc} \frac{\eta^{2}\left(X,Y\right)}{\sqrt{\eta^{2}\left(X\right)\cdot \eta^{2}\left(Y\right)}}, & \mathrm{if}\;\eta^{2}\left(X\right)\cdot \eta^{2}\left(Y\right)>0;\\ 0, & \mathrm{otherwise}. \end{array}\right.$$

Next, we describe the DisCo P-ad algorithm as used in our context with *M* features (metabolites’ abundances) and *n* mutually independent samples (patients).

1. Compute DisCo for each of the M2 pairs of metabolites, where each metabolite abundance (log-transformed and adjusted for covariate(s), if applicable) is a univariate random variable. To compute DisCo, we used the computationally efficient *fastDcov()* function [29] provided in Chaudhuri and Hu [30].
2. Check whether the M×M empirical DisCo matrix is positive definite. If not, convert the empirical DisCo matrix to the nearest (we chose with respect to the Frobenius norm) non-negative definite matrix [31] using the *nearestSPD()* function in [29]. We set all eigen-values less than $10^{-12}$ to zero.

Note that the DisCo P-ad algorithm can be applied to any of the eigen-analysis-based methods in Section 2.1.

### 2.5. Real Data Application

We applied the DisCo P-ad and PrsCo P-ad (Pearson-correlation-based *p*-value adjustment) to a liquid chromatography–mass spectrometry (LC–MS) metabolomics dataset from the COPDGene cohort obtained using the Metabolon platform [32] for studying chronic obstructive pulmonary disease (COPD). The COPDGene Visit 2 dataset is generated from the plasma samples of 1136 individuals, containing abundances of 1005 metabolites on each sample. We used the imputed and normalized dataset for our analysis and omitted the 2 partially characterized molecules (compound IDs: 62145 and 62146; chemical IDs: 100020253 and 100020254) and 242 unidentified metabolites from subsequent analyses. Thus, the total number of metabolites retained was M=761. Among the 1136 total number of subjects, we omitted 82 who had missing data on the measure of a patient’s cigarette-smoking history as published by *American Thoracic Society* (ATS) journals (ATS pack-years) covariate and the ratio of post-bronchodilator forced expiratory volume at one second (FEV_1_) to forced vital capacity (FVC) outcome [33], thus eventually using the remaining 1054 subjects for the statistical analysis. The COPDGene data are available at the National Institutes of Health (NIH) Common Fund’s National Metabolomics Data Repository, Metabolomics Workbench https://www.metabolomicsworkbench.org ([32]; Study ID ST001443, accessed on 7 January 2025) with data-processing described in [34].

As an example of an analysis that may be performed, we tested for the association of sex (female/male) [35] with metabolite abundances using the Welch’s two-sample parametric *t*-test [36], without any covariate adjustment. To explore two different sample sizes, we stratified on two sub-groups based on disease severity defined using the Global Initiative for Chronic Obstructive Lung Disease (GOLD) recommendations: (1) control COPD subjects (GOLD value = 0; n=448) and (2) subjects with *severe* and *very severe* COPD (GOLD values = 3 or 4; n=183). For notational convenience, we refer to the latter group simply as ”severe COPD” throughout the rest of the article.

As an initial quick check, we examined the visualization performance of the t-SNE algorithm [37,38] in revealing any obvious grouping of patients by sex as explained by the abundances of metabolites. No notable results were found for either of the two COPD GOLD subpopulations (see Supplement Figure S3a,b). Next, we compared the eigen-analysis-based estimated PWERs (and ${\hat{M}}_{\mathrm{eff}}$s) with those obtained from the permutation-based gold values (number of permutations, K=10,000) to draw conclusions about the efficacy of the PrsCo- and DisCo-based approaches. Note that we only considered the ungrouped and the defined metabolites’ groupings scenarios for this application (Section 2.6).

### 2.6. Grouping of the Metabolites

We first considered all the M=761 metabolites individually (ungrouped). However, metabolites can be grouped into pathways based on the Metabolon platform annotation. To investigate the effect of pathway-based grouping of the metabolites on the performance of eigen-analysis-based ${\hat{M}}_{\mathrm{eff}}$ computations, we considered the following two types of grouping:

1. Defined grouping: Based on the available annotation information on Metabolon super-pathways, we grouped the metabolites into the following eight categories (number of metabolites in parentheses): lipid (365), xenobiotics (99), amino acid (181), carbohydrate (24), cofactors and vitamins (25), nucleotide (32), energy (10), and peptide (25).
2. Randomly assigned: Keeping the number of groups and the number of members in each group unchanged as above, we randomly assigned the M=761 metabolites into 8 groups.

### 2.7. Simulation Setup

We varied the following parameters in our simulations:

1. Sample size (n): “small”, n=100 (≈10% of total *n*; n:M≈1:7.6), “moderate”, n=500 (≈50% of total *n*; n:M≈1:1.5), and “large”, n=1000 (≈100% of total *n*; n:M≈1:0.76). Note that, for each of these scenarios, we had a fixed number of metabolites, i.e., M=761. For each of the simulation repetitions, we sub-sampled, from the total pool of n=1054 subjects in the COPDGene dataset, a data subset of sample size n=100,500, or 1000, to generate data from a multivariate Gaussian distribution. The mean-vectors and the covariance matrices were estimated from the corresponding sub-sampled data subsets [10]. Based on these simulated data, we then computed the permutation-based gold-standard ${\hat{M}}_{\mathrm{eff}}^{0}$’s and those for each of the eigen-analysis-based methods (${\hat{M}}_{\mathrm{eff}}$’s).
2. Grouping of metabolites (see Section 2.6):
  (a) Ungrouped: We used all the M=761 metabolites together for the eigen-analysis-based methods.
  (b) Defined: We divided the M=761 metabolites into 8 groups based on pathway annotation.
  (c) Random: We divided the M=761 metabolites randomly among the 8 groups, maintaining the same cardinality of groups as for defined grouping.
  Note that for the two grouped scenarios above, we estimated the effective number of tests separately for each sub-group and then added those to obtain the final ${\hat{M}}_{\mathrm{eff}}$ value.
3. Nature of the outcome: As a continuous outcome, we used the logit-transform of the ratio FEV_1_/FVC [33,34]. Before performing the analysis, we adjusted for the effects of the following six fixed covariates on the log-abundance of each of the 761 metabolites and used the residuals as the adjusted metabolite concentrations: age (in years), body mass index (BMI), ATS pack-years, sex (female/male), smoking status (former/current), and data collection center (National Jewish Center (NJC)/University of Iowa (UIA)). For an example of a binary outcome, we considered a subject’s sex (female/male) as an outcome of interest since metabolite features may be strongly predictive of sex [35]. The metabolite abundances were adjusted for the same covariates as above, except for sex.

### 2.8. Summary Metric for Performance Evaluation

We summarized the simulation results, computing the medians of the estimated M_eff_’s across the 100 simulation repetitions (random subsets from the COPDGene data; see Section 2.7). As a metric for the estimation performance, we reported the root-mean-squared error (RMSE) of the ${\hat{M}}_{\mathrm{eff}}$’s, where the “true” values were considered those obtained from the corresponding permutation-based gold-standards ${\hat{M}}_{\mathrm{eff}}^{0}$. We applied the PrsCo P-ad and the DisCo P-ad for all five eigen-analysis-based procedures under comparison. The smaller the RMSE, the better the performance.

### 2.9. Computing Software

Most of the results and figures were generated using R (v. 4.1-4.4; [39]). The distance correlations were computed using MATLAB (v. 9.14.0.2206163 (R2023a); [40]). The permutation-based gold-standard for the simulations were computed using the high-performance cluster computing facilities at the Research Computing unit of the University of Colorado Boulder (https://www.colorado.edu/rc/; accessed on 7 January 2025).

## 3. Results

We used simulations and a real-data application to compare the performance of PrsCo P-ad and DisCo P-ad for five different eigen-analysis-based M_eff_ estimating methods (see Section 2.1), alongside the Bonferroni and Šidák corrections, to the permutation-based gold-standard values. Note that the permutation gold values used the outcome for regression modeling, whereas none of the other methods did to estimate M_eff_.

### 3.1. Gold-Standard ${\hat{M}}_{eff}$s

With increasing *n*, the median ${\hat{M}}_{\mathrm{eff}}^{0}$ values decreased (i.e., increased ${\hat{\alpha }}_{f}^{0}$), most likely because a higher sample size was able to better capture the null association among the metabolite abundances and the outcome (Table 1, row 1). As expected, the rate of this decrease reduced for higher *n*’s (e.g., from n=500 to n=1000). These gold-standard ${\hat{M}}_{\mathrm{eff}}^{0}$ values were used for evaluating the performance of the eigen-analysis-based methods.

### 3.2. Continuous Outcome

We estimated the DisCo P-ad- and the PrsCo P-ad-based $M_{\mathrm{eff}}$ values using the metabolite abundances adjusted for the six clinical covariates (Section 2.7).

The ${\hat{M}}_{\mathrm{eff}}$’s for Bonferroni and Šidák were, respectively, 761 and 742, irrespective of the sample sizes and types of grouping of metabolites. These values were higher than the corresponding gold-standard ones, leading to more conservative PWERs, resulting in potentially false negatives. The primary comparison was between DisCo and PrsCo, but we also compared performance across the five eigen-analysis-based methods and the three types of metabolite groupings.

For the smallest sample size scenario (n=100), DisCo-based results outperformed the corresponding PrsCo-based ones for all the eigen-analysis-based methods and the three metabolite groupings. For the moderate sample size scenario (n=500), DisCo-based results also outperformed the corresponding PrsCo-based ones for all the eigen-analysis-based methods, except for Nyholt in the defined and random grouping case, and Gao for the random grouping case. However, in these three exceptions, the estimated effective number of tests were close between PrsCo and DisCo (within five). For the largest sample size scenario (n=1000), DisCo-based results again met or outperformed the corresponding PrsCo-based ones, except for a few exceptions (Gao for ungrouped, Peluso for ungrouped and defined groupings, and Nyholt for defined and random groupings). In 3 of these 5 exceptions, the differences between PrsCo and DisCo were close (within 12).

**Figure 1:**
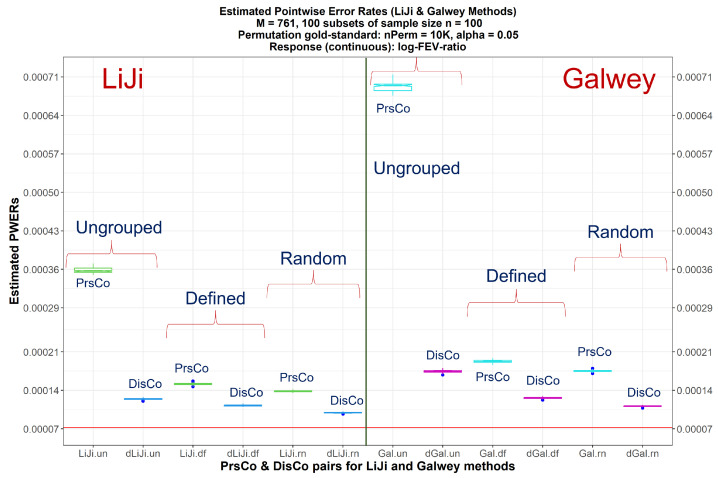
Boxplots of estimated PWERs (across 100 repetitions) obtained by LiJi and Galwey methods using random subsets of sizes of n=100 from the COPDGene dataset. The red horizontal line corresponds to the median permutation-based gold-standard PWER value at 5% level of significance for the continuous outcome: logit of FEV_1_/FVC ratio. The first 6 boxplots from the left correspond to the LiJi method and the rightmost 6 correspond to the Galwey method. For each of the two procedures, the 6 boxplots indicate the estimated PWERs for PrsCo/DisCo pairs, respectively, for ungrouped, defined grouping, and random grouping of p=761 metabolites. PrsCo: LiJi—green, Galwey—cyan; DisCo: LiJi—blue, Galwey—magenta. The closer the boxplots are to the red horizontal line, the better the performance of the concerned method. Gal: Galwey method, un: metabolites ungrouped, df = defined metabolites’ grouping, rn = random metabolites’ grouping.

As a secondary comparison, the PWERs are better estimated using DisCo compared to PrsCo for the LiJi and Galwey methods, varying sample sizes (n=100,500, and 1000) and metabolites grouping (Figure 1 and Figure S1a,b). Similar figures for all three sample sizes and three metabolite groupings but with all the seven methods are provided as supplementary materials (see Figure S2a–i).

Comparing the different methods using DisCo, for n=100 and all three metabolite groupings, the smallest RMSE was achieved by the Nyholt method, outperforming all the other methods by large margins. For the larger sample sizes, Gao and Peluso had the smallest RMSEs using DisCo, especially for ungrouped and defined grouping scenarios. Notably, some methods estimated M_eff_’s higher than the gold-standard one, which means that the corresponding PWER’s (significance level α divided by ${\hat{M}}_{\mathrm{eff}}$) will be more conservative, leading to potential false negatives (but fewer than those produced by Bonferroni and Šidák, except for Peluso in random grouping). In contrast, other methods produced smaller than gold-standard ${\hat{M}}_{\mathrm{eff}}$’s, leading to more lenient PWERs, i.e., potentially higher false positives. In general, Nyholt for all sample sizes tended to have estimates higher than the gold standard. Peluso tended to have higher estimates for the larger sample sizes (n=500,1000) and Gao for moderate sample size (n=500) with random grouping and for the largest sample size (n=1000) with both defined and random groupings.

Comparing the effect of groupings of metabolites using DisCo, for almost all sample sizes and methods, either defined or random groupings had the smallest RMSEs compared to the ungrouped scenario. For Nyholt, ungrouped and random performed similarly and worse (larger RMSE) than the defined grouping results. In the LiJi and the Galwey methods, the RMSEs decreased from ungrouped to defined to random groupings. Finally, for both Gao and Peluso, for smaller sample size (n=100), random grouping performed better compared to ungrouped; while for larger sample sizes (n=500 or 1000), defined grouping performed better.

### 3.3. Categorical Outcome

Unlike the continuous outcome, for sample sizes smaller than the number of metabolites (n=100 and 500), the permutation-based gold-standard PWERs did not give rise to the desired FWER (α=0.05). We, therefore, included the sole scenario of the largest sample size (n=1000) in the results (Table 2). By definition, the ${\hat{M}}_{\mathrm{eff}}$’s for Bonferroni and Šidák are again, respectively, 761 and 742, irrespective of the types of grouping of metabolites. Here, again, these values are higher than the gold-standard leading to more conservative PWERs, resulting in potentially false negatives. The primary comparison was between DisCo and PrsCo, but we also compared performance across the five eigen-analysis-based methods and the three types of metabolite groupings.

Across all the metabolite groupings, DisCo-based results outperformed the corresponding PrsCo-based ones for LiJi, Gao, and Galwey methods. For Nyholt, DisCo- and PrsCo-based estimated effective number of tests were close (within 6), while for Peluso, PrsCo-based results differed with larger margins for ungrouped and defined grouping scenarios (minimum difference 46), quite similar to the case for the continuous outcome.

Comparing the different methods using DisCo, across all the metabolite grouping scenarios, the smallest RMSE was jointly achieved by the Gao method (ungrouped), the LiJi method (random), and the Galwey method (random), outperforming all the other methods by large margins. Once again, the enhanced performance of Gao was prominent for a larger sample size compared to the number of features. Please note that, except for LiJi and Galwey (all groupings) and for Gao (ungrouped), all other methods estimated M_eff_’s much higher than the gold-standard one, which means that the corresponding PWERs (significance level α divided by ${\hat{M}}_{\mathrm{eff}}$) will be more conservative, leading to potential false negatives (but often fewer than those produced by Bonferroni and Šidák, except for Peluso in ungrouped and random grouping). In contrast, for the above-mentioned exclusions, the ${\hat{M}}_{\mathrm{eff}}$’s will lead to more lenient PWERs, i.e., potentially higher false positives. Peluso tended to have higher estimates (>M=761) for the random grouping scenario.

Comparing the effect of groupings of metabolites using DisCo for different methods, similar to the continuous outcome case, the smallest RMSEs corresponded to defined or random grouping compared to ungrouped, except for Gao (best RMSE for ungrouped). Nyholt, for ungrouped and random groupings, performed similarly and worse (larger RMSE) than the defined grouping results. For LiJi and Galwey methods, the RMSEs decreased from ungrouped to defined to random groupings. For both these methods, the best performance (smallest RMSE) corresponded to the random grouping (and not defined grouping). Finally, for Peluso, defined grouping performed the best compared to ungrouped and random scenarios.

### 3.4. Real Data Application

In Table 3 and Table S1, we present the results for the two strata—severe COPD (GOLD = 3 or 4) and controls (GOLD = 0), respectively—considering a subject’s sex as the binary outcome and the individual log-metabolite abundances as the continuous predictors (without any covariate adjustment), using the parametric Welch’s two-sample *t*-test. All the PWERs are computed for a preset FWER of α=0.05.

For severe COPD cases (n=183) and ungrouped metabolites scenario, the DisCo-based ${\hat{M}}_{\mathrm{eff}}$’s (and, hence, corresponding PWERs) were closer to the gold-standard for all methods (smaller for Nyholt and higher for the rest). Maximum improvement was noticed for Gao, followed by Peluso, then Galwey, and LiJi methods. Consequently, the number of significant metabolites for these methods were, respectively, 11, 10, 9, and 7 fewer compared to what were found using PrsCo, potentially eliminating some false positives. For Nyholt, although DisCo performed better (${\hat{M}}_{\mathrm{eff}}$ less by 7), no effect was reflected in significant metabolite findings. For the defined metabolite grouping scenario, DisCo-based ${\hat{M}}_{\mathrm{eff}}$’s continued to be closer to the gold standard for all methods except Gao (higher for LiJi, Galwey, and Peluso and smaller for Nyholt), resulting in four significant metabolites fewer than the PrsCo-based ones for LiJi and Galwey, thus potentially eliminating some false positives. PrsCo and DisCo ${\hat{M}}_{\mathrm{eff}}$’s for Nyholt stayed very close (difference of two), with no effect found in significant metabolite findings. Finally, the DisCo-based ${\hat{M}}_{\mathrm{eff}}$ for Peluso led to only one fewer significant metabolite compared to the PrsCo-based finding.

For COPD controls (n=448) and ungrouped metabolites scenario, the DisCo-based ${\hat{M}}_{\mathrm{eff}}$’s (and, hence, corresponding PWERs) were higher and closer to the gold-standard one for all methods except Peluso. Maximum improvement was noticed for Gao and Galwey methods. Consequently, for both these methods, the number of significant metabolites was eight fewer compared to what was found using PrsCo, potentially eliminating some false positives. For Peluso, the PrsCo-based results appeared to be smaller and closer to the gold-standard with five significant metabolites higher than the DisCo-based result. For Nyholt, PrsCo and DisCo performed almost similar (${\hat{M}}_{\mathrm{eff}}$ difference of two) with no effect in significant metabolite findings. For the defined metabolite grouping scenario, DisCo-based ${\hat{M}}_{\mathrm{eff}}$’s continued to be higher and closer to the gold standard for LiJi and Galwey methods, resulting in, respectively, four and three significant metabolites fewer than the PrsCo-based ones, thus potentially eliminating some false positives. PrsCo and DisCo ${\hat{M}}_{\mathrm{eff}}$’s for Gao and Nyholt were almost similar (maximum difference of seven), with no difference in significant metabolite findings. Finally, the PrsCo-based ${\hat{M}}_{\mathrm{eff}}$ for Peluso was smaller and closer to the gold standard with only one additional significant metabolite.

## 4. Discussion and Concluding Remarks

Adjustment procedures for *p*-values in multiple testing for high-dimensional correlated data have primarily been focused on GWAS applications to account for SNP structure. Less has been studied in other omics contexts, such as metabolomics, where the associations between features may have different characteristics. We introduced DisCo P-ad as an alternative approach based on the distance correlation that is more flexible to account for linear and non-linear associations that may occur between metabolites. We also considered the groupings of metabolites in pathways in the adjustment procedure. We demonstrated the effectiveness of DisCo P-ad via numerical examples applied to the COPDGene metabolomics dataset. Our simulations and real data application confirmed the enhanced performance of DisCo P-ad compared to PrsCo P-ad (its Pearson counterpart), for both continuous and categorical (binary) outcomes, varying ratios of sample sizes (*n*) to the number of predictors (*M*; metabolite abundances), and defined and random grouping of metabolites.

DisCo P-ad can be used on different adjustment methods with varying effects. For the Nyholt procedure, the DisCo-based ${\hat{M}}_{\mathrm{eff}}$ can potentially reduce false negatives, while for LiJi, Gao, Galwey, and Peluso, it can help reduce false positives. For the LiJi and Galwey procedures, DisCo P-ad yielded better results for all simulation scenarios and the real data application. The benefits of DisCo P-ad was more pronounced for smaller n/M ratios for the Nyholt procedure. Similarly, for Peluso, which does not guarantee ${\hat{M}}_{\mathrm{eff}}\leq M$ for moderate to large n/M ratios, DisCo also performed better for smaller n/M ratios.

To compare the particular effect of DisCo, we also used the correlation matrix of another non-linear, non-parametric correlation measure—Spearman’s correlation [41]—especially for the small-sample-size scenario (n=100), where the DisCo outperformed the PrsCo with the highest margins. However, the results with Spearman correlation were relatively similar to those obtained from PrsCo.

Grouping metabolites also provided some improvement in performance. Generally, the ${\hat{M}}_{\mathrm{eff}}$’s became closer to the gold-standard values for defined groupings of metabolites based on pathway information or random groupings compared to those for the ungrouped case. One reason could be that the estimation of M_eff_’s was more accurate when we leveraged the intrinsic association structure among the metabolites. The random grouping may still be capturing metabolite pathway structure.

For grouping the features, instead of hard grouping (as we did for the metabolites based on the super-pathway information), one may technically implement some data-driven method, such as hierarchical clustering [42], to allow for flexible hierarchical structure among the features. However, this may result in an increase in computation time, depending on the number of parameters involved to optimize the hierarchical clustering.

The real data analysis illustrates example applications of identifying differentially abundant metabolites using two different sample sizes. Especially with the smaller sample size strata (severe COPD), we observed the benefits of the DisCo approach compared to PrsCo for almost all of the eigen-analysis-based methods and grouping types. For the larger sample size strata (controls), the differences were less pronounced, but DisCo also showed a benefit for a majority of the combinations.

Note that the proposed DisCo-based approach outperformed the PrsCo for all scenarios, especially when the sample size was small, i.e., n=100, possibly due to Pearson correlation being more influenced by noise or outliers than the more robust distance correlation. We also note that with a more high-dimensional dataset (larger number of predictors), the benefit of the DisCo-based approach may be more pronounced even for larger sample sizes. However, there is relatively less exploration of the effective number of tests for metabolomics data, so this work is more focused on the typical number of predictors found in metabolomics studies. Exploring scenarios with the larger number of predictors typically found in genetic and epigenetic studies is a topic for future work.

Next, note that, prior to the eigen-analysis step, the approximation of the original correlation matrices (PrsCo and DisCo) to their nearest positive-definite matrices using the R function *nearPD()* and the MATLAB function *nearestSPD()*, respectively, may “distort” the original dependencies. Ideally, these approximations have to be tested in different scenarios, especially in cases where the original matrices are far from the SPD versions.

To obtain the eigen-solution, DisCo requires the computation of the distance correlation matrix, while PrsCO can rely on singular value decomposition of the standardized data, leading to improved computational memory and efficiency. While the relatively greater computation cost for DisCo compared to PrsCo is tolerable with cutting-edge computing resources, the memory allocation of the precomputed *M*-by-*M* DisCo matrix might prove to be prohibitive when *M* is on the order or millions or more. An area of future work is to explore whether singular value decomposition would be applicable for the DisCo matrix as well. An interested reader can refer to [43] for a comparative study on PrsCo and DisCo.

In summary, while our focus for this article was to investigate the efficacy of DisCo P-ad for metabolomics data, one can readily apply our proposed procedure to eigen-analysis for other types of omics data as well. In addition, DisCo P-ad can be applied for multiple testing corrections in multi-omics studies where data are integrated from different “layers” of understanding (e.g., genomics, transcriptomics, proteomics, microbiomics, metabolomics, etc. [1]) to discover molecular pathways not possible by examining a single layer only.

## Figures and Tables

**Table 1:** Simulations for Continuous Outcome (n=100,500, and 1000). All simulations were repeated 100 times and the medians of the estimated effective number of tests for logit of FEV_1_/FVC outcome are reported. The first three rows contain the permutation-based gold-standard medians of the estimated effective number of tests, along with the standard correction methods of Bonferroni and Šidák. For the gold-standard values, in parentheses are the median absolute deviations. For all other rows, in parentheses are the root-mean-squared errors (RMSE’s), computed as a measure of deviation from the corresponding permutation-based gold-standard values. All numbers are rounded off to the nearest integers. Sub-tables correspond to varying sample sizes (across columns; n=100,500, and 1000) and varying metabolites’ grouping (across rows; ungrouped, defined by super-pathways, and random). Rows correspond to the five eigen-analysis-based procedures. The smaller the RMSE values, i.e., the closer the estimated M_eff_’s are to the corresponding gold-standard values, the better the performance. Cell-values in bold indicate when DisCo P-ad performed better than the corresponding PrsCo P-ad counterpart, decided based on their respective RMSE’s.

Sample Size	*n* = 100		*n* = 500		*n* = 1000	
Permutation-based gold-standard	694 (37)		632 (27)		611 (33)	
Bonferroni	761 (74)		761 (132)		761 (152)	
Šidák	742 (58)		742 (114)		742 (133)	
	Metabolites’ Grouping: Ungrouped					
	PrsCo	DisCo	PrsCo	DisCo	PrsCo	DisCo
Nyholt	739 (55)	**725 (44)**	745 (117)	**744 (116)**	**746 (137)**	**746 (137)**
LiJi	140 (556)	**402 (294)**	303 (330)	**417 (216)**	331 (282)	**419 (195)**
Gao	91 (604)	**416 (280)**	397 (236)	**511 (124)**	**546 (70)**	534 (82)
Galwey	72 (623)	**287 (409)**	241 (392)	**352 (281)**	316 (296)	**366 (247)**
Peluso	155 (541)	**528 (170)**	522 (113)	**734 (108)**	**686 (81)**	786 (176)
	Metabolites’ Grouping: Defined					
	PrsCo	DisCo	PrsCo	DisCo	PrsCo	DisCo
Nyholt	721 (42)	**712 (37)**	**727 (99)**	731 (103)	**728 (119)**	733 (125)
LiJi	330 (365)	**445 (252)**	409 (224)	**465 (169)**	421 (192)	**468 (146)**
Gao	354 (342)	**553 (145)**	613 (34)	**621 (30)**	646 (44)	**630 (32)**
Galwey	260 (436)	**396 (300)**	407 (226)	**453 (181)**	429 (185)	**461 (152)**
Peluso	352 (346)	**486 (212)**	572 (66)	**626 (29)**	**605 (28)**	652 (49)
	Metabolites’ Grouping: Random					
	PrsCo	DisCo	PrsCo	DisCo	PrsCo	DisCo
Nyholt	736 (53)	**725 (45)**	**741 (113)**	744 (116)	**742 (133)**	746 (137)
LiJi	362 (334)	**504 (194)**	469 (165)	**536 (100)**	487 (128)	**540 (77)**
Gao	361 (335)	**584 (115)**	**650 (34)**	655 (37)	684 (77)	**665 (60)**
Galwey	286 (410)	**450 (247)**	472 (161)	**524 (111)**	500 (115)	**535 (81)**
Peluso	486 (211)	**593 (107)**	835 (206)	**821 (193)**	890 (280)	**876 (266)**

**Table 2:** Simulations for sex outcome (n=1000). All simulations were repeated 100 times and the medians of the estimated effective number of tests are reported. The first three rows contain the permutation-based gold-standard median estimated effective number of tests, along with the standard correction methods of Bonferroni and Šidák. For the gold-standard value, in parentheses is the median absolute deviation. For all other rows, in parentheses are the root-mean-squared errors (RMSEs), computed as a measure of deviation from the corresponding permutation-based gold-standard value. All numbers are rounded off to the nearest integer. Sub-tables correspond to varying metabolites’ grouping (ungrouped, defined by super-pathways, and random) and rows correspond to the five eigen-analysis-based procedures. The smaller the RMSE values, i.e., the closer the estimated M_eff_’s are to the gold-standard values, the better the performance. Cell values in bold indicate when DisCo P-ad performed better than the corresponding PrsCo P-ad counterpart, decided based on their respective RMSE’s.

Permutation-Based Gold-Standard	529 (23)					
Bonferroni	761 (230)		761 (230)		761 (230)	
Šidák	742 (211)		742 (211)		742 (211)	
	Metabolite Grouping Ungrouped		Metabolite Grouping Defined		Metabolite Grouping Random	
	PrsCo	DisCo	PrsCo	DisCo	PrsCo	DisCo
Nyholt	**746 (215)**	**746 (215)**	**727 (196)**	733 (202)	**742 (211)**	746 (215)
LiJi	328 (206)	**412 (123)**	417 (118)	**463 (73)**	485 (53)	**537 (24)**
Gao	545 (27)	**530 (24)**	645 (115)	**627 (98)**	683 (152)	**661 (130)**
Galwey	313 (221)	**362 (173)**	426 (109)	**458 (79)**	496 (43)	**530 (24)**
Peluso	**679 (149)**	774 (243)	**599 (70)**	645 (115)	887 (356)	**867 (335)**

**Table 3:** Real-data application results: severe COPD (GOLD = 3 or 4) sub-population, n=183. Estimated effective number of tests (M_eff_’s), point-wise error-rates (PWERs), and number of significant metabolites based on the parametric Welch’s two-sample *t*-test, for ungrouped and defined metabolites’ grouping, corresponding to the permutation-based gold standard and all five eigen-analysis-based methods are reported. Numerical values in bold within ${\hat{M}}_{\mathrm{eff}}$ and estimated PWER cells indicate those that are closer to the gold-standard permutation ones. Values within parentheses for ${\hat{M}}_{\mathrm{eff}}$ indicate the absolute differences from the gold-standard. Bold for the numbers of significant metabolites indicate that the PrsCo-based and the DisCo-based findings differ.

GOLD = 3 or 4 (n=183)		${\hat{M}}_{\mathrm{eff}}$	Estimated PWER	*t*-Test: No. of Significant Mets	${\hat{M}}_{\mathrm{eff}}$	Estimated PWER	*t*-Test: No. of Significant Mets
Permutation		531	9.42 $\times 10^{-05}$	-	531	9.42 $\times 10^{-05}$	-
		Metabolites: Ungrouped			Metabolites: Defined Grouping		
Nyholt	PrsCo	742 (211)	6.74 $\times 10^{-05}$	37	724 (193)	6.91 $\times 10^{-05}$	37
	DisCo	**735 (204)**	**6.80 $\times 10^{-05}$**	37	**722 (191)**	**6.93 $\times 10^{-05}$**	37
LiJi	PrsCo	220 (311)	2.27 $\times 10^{-04}$	**50**	371 (160)	1.35 $\times 10^{-04}$	**45**
	DisCo	**408 (123)**	**1.23 $\times 10^{-04}$**	**43**	**449 (82)**	**1.11 $\times 10^{-04}$**	**41**
Gao	PrsCo	172 (359)	2.91 $\times 10^{-04}$	**52**	**480 (51)**	**1.04 $\times 10^{-04}$**	**41**
	DisCo	**455 (76)**	**1.10 $\times 10^{-04}$**	**41**	585 (54)	8.55 $\times 10^{-05}$	**39**
Galwey	PrsCo	122 (409)	4.10 $\times 10^{-04}$	**55**	332 (199)	1.51 $\times 10^{-04}$	**46**
	DisCo	**312 (219)**	**1.60 $\times 10^{-04}$**	**46**	**421 (110)**	**1.19 $\times 10^{-04}$**	**42**
Peluso	PrsCo	261 (270)	1.92 $\times 10^{-04}$	**49**	455 (76)	1.10 $\times 10^{-04}$	**41**
	DisCo	**607 (76)**	**8.24 $\times 10^{-05}$**	**39**	**545 (14)**	**9.17 $\times 10^{-05}$**	**40**

## Data Availability

The original COPDGene data presented in the study are publicly available at: Metabolomics Workbench–Study ID ST001443 (accessed on 7 January 2025). Furthermore, the source codes and the RData files used for analyses are publicly available at the Kechris Lab Git repository (accessed on 7 January 2025). Supplementary Materials are submitted to the journal.

## Supplementary Materials

The following supporting information can be downloaded at: https://doi.org/10.5281/zenodo.14057773, 1. Simulation: Continuous Outcome - Additional Results: (a) Figure S1: Boxplots of estimated PWER’s (across 100 repetitions) obtained by LiJi and Galwey methods using random subsets of sizes of n=500 (a) and n=1000 (b) from the COPDGene data set. (b) Figure S2: Continuous outcome: logit of FEV_1_/FVC ratio: Boxplots of estimated PWER’s (across 100 repetitions) obtained by Bonferroni, Šidák, Nyholt, LiJi, Gao, Galwey, and Peluso methods using random subsets of sizes of n=100 (a–c), n=500 (d–f), and n=1000 (g–i) from the COPDGene data set. For each triplet, the sub-figures correspond to the three types of metabolites’ grouping: ungrouped—(a,d,g); defined—(b,e,h); and random—(c,f,i). 2. Real-Data Application: Results for COPD controls (GOLD = 0) and severe COPD (GOLD = 3 or 4): (a) Figure S3: 2-D t-SNE scatterplots of COPD GOLD = 0 (a) and GOLD = 3 or 4 (b) subpopulations, as explained by 761 log-metabolites abundances (unadjusted for covariates). For each subpopulation, the patients are color-coded by their sex. (b) Table S1: Real-data application results: COPD control (GOLD = 0) sub-population, n=448. Estimated M-eff’s, point-wise error-rates (PWER’s), and number of significant metabolites based on the parametric Welch’s two-sample t-test, for ungrouped and defined metabolites’ grouping, corresponding to the permutation-based gold-standard and all 5 eigen-analysis based methods.

## Abbreviations

The following abbreviations are used in this manuscript:

GWAS	Genome-Wide Association Studies
MWAS	Metabolome-Wide Association Studies
DisCo P-ad	Distance-Correlation-Based P-value Adjustment
FWER	Family-Wise Error Rate
PWER	Point-Wise Error Rate
FDR	False Discovery Rate
SNP	Single Nucleotide Polymorphism
PrsCo	Pearson Correlation
PC	Principal Component
PCA	Principal Component Analysis
LC-MS	Liquid Chromatography–Mass Spectrometry
COPD	Chronic Obstructive Pulmonary Disease
NIH	National Institutes of Health
GOLD	Global Initiative for Chronic Obstructive Lung Disease
FEV_1_	Forced Expiratory Volume in one second
FVC	Forced Vital Capacity
ATS	American Thoracic Society
ATS pack-years	Measure of a patient’s cigarette-smoking history as published by the *American Thoracic Society* (ATS) journals
BMI	Body Mass Index
NJC	National Jewish Center
UIA	University of Iowa
RMSE	Root-Mean-Squared Error
